# Myocilin mutations in black South Africans with POAG

**Published:** 2011-04-27

**Authors:** Benjamin T. Whigham, Susan E.I. Williams, Yutao Liu, Robyn M. Rautenbach, Trevor R. Carmichael, Joshua Wheeler, Ari Ziskind, Xuejun Qin, Silke Schmidt, Michele Ramsay, Michael A. Hauser, R. Rand Allingham

**Affiliations:** 1 Center for Human Genetics, Duke University Medical Center, Durham, NC; 2 Division of Ophthalmology, Department of Neurosciences, University of the Witwatersrand, Johannesburg, South Africa; 3 Division of Ophthalmology, Department of Surgical Sciences, Stellenbosch University, Cape Town, South Africa; 4 Division of Human Genetics, NHLS and School of Pathology, University of the Witwatersrand, Johannesburg, South Africa; 5 Department of Ophthalmology, Duke University Eye Center, Durham, NC

## Abstract

**Purpose:**

Myocilin (*MYOC*) mutations are associated with primary open-angle glaucoma (POAG) in multiple populations. Here we examined the role of *MYOC* mutations in a black South African population with primary open-angle glaucoma (POAG).

**Methods:**

Unrelated black South African subjects with POAG and unaffected controls were recruited from the St. John Eye Hospital (Soweto, Johannesburg, South Africa) and East London Hospital Complex (Eastern Cape, South Africa). A complete eye examination including visual field assessment was performed in all subjects. Blood samples were obtained for DNA extraction. The complete coding region of *MYOC* was sequenced using the PCR-based Sanger method. Identified mutations were compared to known *MYOC* mutations.

**Results:**

One hundred-thirteen POAG cases and 131 controls were recruited for analysis. A total of 19 variants were observed. Probable glaucoma-causing mutations were observed in 4.4% of POAG cases. A previously reported glaucoma-causing mutation, Tyr453MetfsX11, was observed in three cases and one control. Two other sequence variants, Gly374Val and Lys500Arg, occurred only in cases. Other sequence variants, including 6 novel variants, occurred in at least one control.

**Conclusions:**

A small minority of black South Africans with POAG carry *MYOC* mutations. The Gly374Val mutation might represent a novel glaucoma-causing mutation. The Tyr453MetFSX11 mutation appears to be a glaucoma-causing mutation with incomplete penetrance.

## Introduction

Glaucoma is a group of disorders that cause progressive loss of retinal ganglion cells, optic nerve cupping, and visual field loss. It is the most common cause of irreversible blindness worldwide [1]. Of glaucoma’s multiple sub-types, the most common is primary open-angle glaucoma (POAG) [1,2]. POAG is currently responsible for an estimated 3.3 million cases of bilateral blindness worldwide [3]. POAG treatment is currently limited to modification of elevated intraocular pressure (IOP), an established risk factor. A greater understanding of other risk factors and molecular mechanisms will likely improve treatment and detection [2].

Genetic risk factors are known to contribute to POAG as first-degree relatives of affected individuals are 3–9 fold more likely to develop the disease [4]. Identification of genetic risk factors could greatly improve detection and treatment of POAG. However, POAG has a complex inheritance pattern that confounds many approaches used to study Mendelian traits. Only a small proportion of POAG cases have been traced to mutations in individual genes. Instead, the majority of POAG cases appear to be influenced or caused by multiple genetic factors [4]. To date, 15 POAG-associated loci have been identified [4]. Within these loci several genes have been identified including myocilin (*MYOC*) [5], optineurin [6],  WD repeat-containing protein 36 (WDR36) [7], and cytochrome p450 1B1 [8]. Among these genes, *MYOC* has been found to harbor more glaucoma-causing mutations than any other identified risk gene [4].

The role of *MYOC* in POAG was first identified through genetic linkage analysis of families carrying juvenile-onset forms of POAG [5,9]. In these families, *MYOC* mutations were found to associate with glaucoma phenotypes characterized by: 1) juvenile or early-age onset (<40 years); 2) high intraocular pressure; and 3) autosomal dominant inheritance. Some *MYOC* mutations are associated with adult-onset (>40 years) POAG and appear responsible for 3%–5% of cases worldwide [2]. Over 70 unique mutations have been identified [10], with many of these mutations being specific to a single population or ethnic group [11].

Given that many *MYOC* mutations are found only in a single population, it is useful to screen *MYOC* in multiple populations. For example, a previous screen of 90 POAG cases from Ghana West-Africa yielded two novel glaucoma-causing mutations that have not been reported in any Caucasians cases [12]. At present, work in numerous populations has led to a large catalog of mutations. This catalog is useful for genetic screening, especially for first-degree relatives of individuals with POAG [10]. The catalog also provides targets for functional studies with mutant MYOC protein. Previous studies have suggested that *MYOC* mutations alter the solubility of MYOC protein [13,14] and disrupt its secretion [15-18]. These studies have benefitted from the growing assortment of mutations observed in humans. Future studies will likely benefit from identifying additional *MYOC* mutations.

Novel *MYOC* mutations will likely be found in sub-Saharan Africa populations. There is more genetic diversity within Africa than anywhere else on Earth [19]. Current data on sub-Saharan Africa is limited to two studies that focused on West Africans. The first was conducted on a Ghanaian cohort in West Africa [12]. The second was conducted on an African American cohort [20], the majority of which were likely of West African ancestry [21]. No studies have been reported in East or South African populations. Here we report the first mutation screen of *MYOC* in black South Africans with POAG. Our study can help to establish the contribution of MYOC mutations to POAG in this population. It also can help identify novel mutations for future functional work and genetic screening.

## Methods

### Study participants

This study adhered to the tenets of the Declaration of Helsinki. The research protocol was approved by all participating universities including the University of the Witwatersrand Human Research Ethics Committee and Stellenbosch University Health Research Ethics Committee. Southern African black participants with clinically diagnosed POAG and unaffected southern African control subjects were recruited from the St. John Eye Hospital in Soweto (Johannesburg, South Africa) and East London Hospital Complex (Eastern Cape, South Africa). Written informed consent was obtained from all participants. Ethnic affiliation was established by the home language of participants and that of their parents and grandparents. All participants underwent a standardized detailed ophthalmic examination by an ophthalmologist (S.E.I.W. or R.R.). The examination included measurement of intraocular pressure (IOP) by applanation, slit lamp biomicroscopy, gonioscopy, dilated pupil examination of the lens and fundus, and visual field testing. Subjects with POAG had evidence of glaucomatous optic neuropathy and visual field loss with open angles on gonioscopy and no evidence for a secondary cause for the glaucoma. Gender- and ethnicity-matched South African subjects with normal anterior segment and optic nerve examination and an IOP of less than 21 mmHg were recruited as control subjects. Control subjects were selected to be older than POAG participants to ensure that a diagnosis of POAG could be excluded in the controls.

### DNA sequencing analysis

Genomic DNA was extracted using standard methodology [22]. Briefly, blood was obtained using peripheral venipuncture.  The blood was anticoagulated and lysed.  Cellular protein was salted out of solution using NaCl.  Genomic DNA, still in solution, was removed, precipitated in ethanol, and resuspended in water. Primers flanking the entire coding sequence of *MYOC* were designed with Primer3 software [23]. Primer sequences are provided in Table 1. The targeted region covered at least 80 base pairs into each intron to screen for potential mutations affecting exon splicing. Platinum Taq DNA polymerase (Invitrogen, Carlsbad, CA) was used for all the polymerase-chain reactions (PCR). The PCR amplifications were performed in ThermoHybaid MBS 02, 02S, and 02G PCR machines (Thermo Scientific, Waltham, MA). Completed PCR reactions were purified and sequenced in the forward direction using BigDye chemistry (Applied Biosystems, Foster City, CA). All the sequences were analyzed using the Sequencher 4.9 software package (Gene Codes, Ann Arbor, MI). Potential mutations were confirmed by repeating PCR amplification with the relevant sample and then sequencing in the reverse direction.

**Table 1:** List of PCR primers for *MYOC* (myocilin) exon sequencing in black South African individuals.

**Myocilin exon**	**Forward primer sequence**	**Reverse primer sequence**	**PCR product size (bp)**	Covered genomic region*
Exon1a	ATCTTGCTGGCAGCGTGAA	TCTCTGGTTTGGGTTTCC	614	chr1:171,621,342–171,621,955
Exon1b	GACAGCTCAGCTCAGGAAGG	GAAGGTGATCGCTGTGCTTT	663	chr1:171,620,991–171,621,653
Exon2	AGCAAAGACAGGGTTTCACC	AGGGCTTTGTTAGGGAAAGG	554	chr1:171,607,517–171,608,071
Exon3a	CCCAGACGATTTGTCTCCAG	TCCCAGGTTTGTTCGAGTTC	648	chr1:171,605,327–171,605,974
Exon3b	GAGAAGGAAATCCCTGGAGC	TGGTGACCATGTTCATCCTTC	598	chr1:171,604,914–171,605,511

The covered genomic regions were based on the February 2009 human reference sequence (GRCh37).

### Statistical analysis

The Fisher’s exact test was used to test the association of variant alleles with POAG. The exact test was also used to examine Hardy–Weinberg equilibrium (HWE) for the observed variants.

## Results

Subject demographics are summarized in Table 2. Briefly, there were 113 POAG cases and 131 control subjects in the study data set. POAG cases had a mean age of 59.6±12.7 years. Controls had a mean age of 70.5±8.7 years. Study subjects were self-identified as black South Africans. Subjects spoke a range of southern Bantu languages including IsiXhosa, IsiZulu, Setswana, Sesotho, Sepedi, Xitsonga, Tshivenda, Siswati, and IsiNdebele. The distribution of languages was similar in cases and controls.

**Table 2:** Demographics of study subjects.

**Demographic**	**Control**		**POAG**	
Age at recruitment	70.5 ± 8.7		59.6 ± 12.7	
**Sex**				
Male	57	44%	55	49%
Female	74	56%	58	51%
**Tribal affiliation**				
IsiXhosa	40	31%	23	20%
IsiZulu	37	28%	41	36%
Setswana	19	15%	15	13%
Sesotho	13	10%	12	11%
Sepedi	6	5%	14	12%
Xitsonga	6	5%	3	3%
Tshivenda	3	2%	3	3%
Siswati	3	2%	1	1%
Isindebele	2	2%	0	0%
Other	2	2%	1	1%

A total of 19 *MYOC* variants were observed in the sampled population. All variants were in HWE (p>0.05). Of the 19 variants, 12 occurred in coding regions, including 7 silent changes, 4 missense changes, and 1 frameshift change (Table 3). Two of these changes appeared to be associated with glaucoma. Tyr453MetfsX11, a frameshift mutation, was observed in 3 POAG cases and 1 control. It was previously reported as a glaucoma-causing mutation [20]. Gly374Val, a novel missense mutation, was observed in two cases and no controls. Neither mutation appeared to cause a clinically distinguishable form of glaucoma when compared to cases that did not carry mutations in *MYOC*. In all, 4.4% of black South African cases carried one of these probable mutations.

**Table 3:** List of coding variants identified from *MYOC* exon sequencing in black South African individuals with or without POAG.

Nucleotide sequence change*	**AA change**	**dbSNP ID**	**Reported pathogenicity**	**Observed in POAG (n=113)**	**Observed in controls (n=131)**
c.1357delT	Tyr453MetfsX11	-	Glaucoma-Causing [20]	3 (2.7%)	1 (0.8%)
c.654G>A	Glu218Glu	-	Novel	2 (1.8%)	3 (2.3%)
c.1121G>T	Gly374Val	-	Novel	2 (1.8%)	0 (0.0%)
c.1054G>A	Glu352Lys	rs61745146	Uncertain [20,24,25]	5 (4.4%)	5 (3.8%)
c.39T>G	Pro13Pro	rs12082573	Neutral [20]	8 (7.1%)	10 (7.6%)
c.227G>A	Arg76Lys	rs2234926	Neutral [20]	0 (0.0%)	1 (0.8%)
c.477A>G	Leu159Leu	rs61730977	Neutral [20]	7 (6.2%)	9 (6.9%)
c.612G>T	Thr204Thr	rs57824969	Neutral [20]	5 (4.4%)	3 (2.3%)
c.975G>A	Thr325Thr	rs61730976	Neutral [20]	14 (12.4%)	6 (4.6%)
c.1041T>C	Tyr347Tyr	rs61730974	Neutral [20]	0 (0.0%)	1 (0.8%)
c.1188G>A	Glu396Glu	rs61730975	Neutral [20]	9 (8.0%)	7 (5.3%)
c.1499A>G	Lys500Arg	-	Neutral [20]‡	2 (1.8%)	0 (0.0%)

*Nucleotides numbered as in Ensembl accession number ENSG00000034971 (transcript ENST00000037502). ‡See text.

Three other protein-altering variants did not appear to be associated with glaucoma. The three variants were Lys500Arg, Arg76Lys, and Glu352Lys. The Lys500Arg substitution has been previously reported and is considered neutral [20]. Here it was observed in 2 cases and no controls. The Arg76Lys substitution has been reported as neutral [20]. We found this variant in no cases and 1 control. Finally, the Glu352Lys substitution has unknown pathogenicity [20,24,25]. We found Glu352Lys in 5 cases and 5 controls.

We identified 14 silent variants in this South African population that were predicted to not impact the amino acid sequence of MYOC. Of these, 7 were synonymous variants in the *MYOC* coding region (Table 3). The other 7 were in intronic or promoter regions (Table 4). All 14 were observed in at least one control. None had been previously reported as glaucoma-causing [20,26]. Among these variants, one significant association was observed (p<0.05). The non-coding variant c.731–73C>T was more common in controls (9.9%) than in cases (1.8%). This variant was observed in 4 tribal groups and was not significantly over- or underrepresented in any one group (p>0.05).

**Table 4:** List of non-coding variants identified from *MYOC* exon sequencing in black South African individuals with or without POAG.

**Nucleotide sequence change**	**SNP ID**	**Reported pathogenicity**	**Observed in POAG (n=113)**	**Observed in controls (n=131)**
c.-92_-91delCT	-	Novel	1 (0.9%)	2 (1.5%)
c.604+13A>C	-	Novel	7 (6.2%)	6 (4.6%)
c.604+50G>A	-	Novel	7 (6.2%)	9 (6.9%)
c.731–73C>T	rs79255460	Novel	2 (1.8%)	13 (9.9%)
c.731–23G>A	-	Novel	2 (1.8%)	1 (0.8%)
c.-83G>A	rs2075648	Neutral [13]	0 (0.0%)	1 (0.8%)
c.730+35A>G	rs2032555	Neutral [19]	10 (8.8%)	21 (16.0%)

*Nucleotides numbered as in Ensembl accession number ENSG00000034971 (transcript ENST00000037502) referenced to the February 2009 human reference sequence (GRCh37).

The interaction of multiple variants within *MYOC* appeared to be an unlikely cause of glaucoma in our cases. Only one person was found to carry multiple nonsynonymous variants. This individual carried Tyr453MetfsX11 and Glu352Lys. The individual had glaucoma, but so did two other participants that carried only Tyr453MetfsX11. All other individuals had no more than 1 nonsynonymous coding variant. Multiple synonymous and non-coding mutations did occur together frequently. However, patterns of linkage disequilibrium among these variants were consistent between cases and controls.

## Discussion

This study represents the first screen for *MYOC* mutations in black South Africans. We observed probable glaucoma-causing mutations in 4.4% of individuals with POAG. This frequency is consistent with previously reported African and non-African cohorts with POAG [2]. Importantly, seven novel variants were observed including a missense mutation, Gly374Val, that was found only in cases.

The novel missense mutation Gly374Val was only observed in cases and appears to cause glaucoma. It occurred in two affected heterozygotes. It was absent in 131 controls. Also, it is predicted to be damaging to the MYOC protein by SIFT [27] and PolyPhen [28]. These lines of evidence suggest that Gly374Val causes POAG.

One other variant, Lys500Arg, was found exclusively in cases. It was observed in two POAG cases that were both heterozygous. Both cases were also heterozygous for a synonymous variant, Glu396Glu, but carried no other variants. Previous evidence regarding the Lys500Arg variant is mixed. Lys500Arg has been observed in POAG-affected individuals in several populations and has not been reported in a control sample [20]. However, it was observed in one sample from the general population by Fingert et al. [20]. Functional predictions on Lys500Arg are conflicting. One group predicted that the Lys500Arg substitution might have structural consequences similar to Ile499Phe [29], a glaucoma-causing mutation [30]. However, Polyphen and SIFT predict Lys500Arg to be tolerated and Ile499Phe to be possibly damaging [27,28]. Lys500Arg is of interest given its close proximity to a cryptic peroxisomal-targeting motif that has been implicated in the pathogenesis of *MYOC*-associated glaucoma [18]. More evidence is needed to establish if Lys500Arg contributes to glaucoma.

The Tyr453MetfsX11 mutation was observed at a relatively high rate in South Africans compared with previously reported populations. It was observed in 2.6% of South African cases (n=113). For comparison, it was observed in 0.3% of African American cases (n=312) [20], and no Ghanaian cases from West Africa (n=90) [12]. This sequence variant has not been reported in any non-African populations.

This study was the first to find Tyr453MetfsX11 in a control. The one unaffected carrier was an 80 year old female with no evidence of glaucoma. Given the identification of an unaffected carrier, the Tyr453MetfsX11 mutation might be pathogenic but incompletely penetrant. Incomplete penetrance has been observed for other known glaucoma-causing mutations such as Gln368stop in Caucasian populations and Arg46stop in Asian populations [11]. Like these mutations, Tyr453MetfsX11 truncates the MYOC protein; however, it is a frameshift mutation that results in the addition of 10 ectopic amino acids. Only a small number of frameshift mutations have been reported to date, and these have been strongly associated with glaucoma [10].

The Glu352Lys variant was also highly represented in this group of black South Africans compared with other populations. We observed the variant in 4.4% of our cases and 3.8% of our controls. By comparison, Glu352Lys has been reported at frequencies of 0.6% in African American (n=312) cases and 0.1% in Caucasian cases (n=727) [20]. These low frequencies in other populations have made classification of the Glu352Lys variant difficult [20,24,25]. However, Glu352Lys appears to be benign in black South Africans as it was observed in several controls. This result is consistent with a previous functional study that found Glu352Lys did not alter MYOC protein solubility like some known pathogenic mutations [13].

It is not clear why an intronic variant, c.731–73C>T, was overrepresented in the controls. One possibility was the variant could be overrepresented in IsiXhosa-speaking subjects, who constituted 31% of control subjects compared to only 20% of POAG subjects (Table 2). However, the IsiXhosa controls carried the variant at a similar frequency (10%) as the total control frequency (9.9%) for this variant. To our knowledge, this variant has not been reported in any previous studies of MYOC. It is listed in the dbSNP database as rs79255460. While it is conceivable that the variant could protect against POAG, it is important to note that a Bonferroni correction would have eliminated significance. Replication of our result would warrant further study of rs79255460.

One challenge with studying POAG in black South Africans is the low rate of diagnosis within this population. A previous study found that, among an urban subpopulation of black adults, only 13% of POAG cases had been diagnosed [31]. The low rate of diagnosis causes problems for both personal disease history and family history. Reported family histories are likely to include many false negatives due to undiagnosed family members. In our study, only one carrier of a *MYOC* mutation claimed to have a parent with glaucoma. The lack of family members with glaucoma may result from historically low rates of diagnosis among black South Africans.

Our results agree with previous work that suggests *MYOC* is not related to population differences in POAG prevalence [20]. Black South Africans, like other African groups, have a high rate of POAG compared to Caucasians [31-34]. However, black South Africans did not have a correspondingly high rate of *MYOC* mutations in this study. Based on this result, it appears that *MYOC* mutations are not responsible for increased levels of POAG in black South Africans. This result is consistent with previous work with African populations [12,20].
